# Supplementary material for: High Molecular Weight Kininogen: A Review of the Structural Literature
Source: Int J Mol Sci. 2021 Dec 13;22(24):13370. doi: 10.3390/ijms222413370 (PMC8706920; doi:10.3390/ijms222413370)
Supplement: Supplementary file 1 [file ijms-22-13370-s001.zip › ijms-1452635-supplementary.pdf]

International Journal of
Molecular Sciences

MDPI

Review

Supplementary materials: High Molecular Weight Kininogen: A review of the structural literature

Michał B. Ponczek [1]

[1] University of Lodz, Faculty of Biology and Environmental Protection, Department of General Biochemistry, Pomorska 141/143, 90-136 Lodz, Poland; michal.ponczek@biol.uni.lodz.pl
* Correspondence: michal.ponczek@biol.uni.lodz.pl; Tel.: ++48426354483

Supplemental Figure S1. Sequence alignment (A) and ancestral relationships of extant vertebrates (B). A) Amino acid sequence alignment of vertebrate high molecular weight kininogens.

```
D5 domain His rich domain is important in contact activation
D6 domain interacts with prekallikrein and Factor XI

Bradykinin-like motif is shown in red
*Amino acid numbering is according to human factor HK.
                                        *1
Human                MKLITILFL---C SRLLLSLTQE -SQSEEIDCND KDLFKAVDAA LKKYNSQNQSN NQFVLYRITE
Dog                  MKLLAMLFL---C SRLLPSLTQE -SLSEEIDCND EDLFKAVDTA LKKYNSRNQSG NQFVLYRVTE
Siberian Tiger       MKLIAVLFL---C SRLLPSLTEE -SFSQDIDCND EDLFKAVDTA LKKYNSRNQSG NQFVLYRVTE
Grizzly Bear         MRLLALLFL---C SRLLPSVSQE -SSQE-IDCDD EDLFKAVDTA LKKYNSRNQIG NQFVLHRVTE
Hippopotamus         MKLITILFL---C SRLLPSLTQD -SLQE-IDCND QDVFEAVDTA LKKYNSGNKSG NQFVLYRVTE
BN Dolphin           MKLITILFL---C SRLLPSLTQD -SSQE-IDCND PDVFQAADTA LKKYNSGNKSG NQFVLYRVTE

Opossum              MKLAVVL-L-L-V TSQL-NVQGE -S-—E-VSCQD NDVFRAMDAA LTEYNNQKTSG NQFVLHQIMA
Koala                MELAAIL-L-L-V A-IQLNVQVV -SQVKDVACDD NDVFQAVDVA LTKYNNQKSSG PQFVLYRIIT
Wombat               MELAVIL-L-L-V AS-QLNVQVE -SQVKDVACDD NDVFRAVDVA LTKYNNQKSSG PQFVLYRIIT

Platypus             MKLLGVL-L-F-L GSSLLPSRTA -PVPQDVDCND SDVFKAVDRA LRWYNEHLKDG NQFLLYRVTE

Kiwi                 MKPFLAI--VL-C CSFFSSRATP -LPFEFSDCDD PDVLEAVDTA LKKYNGGRTTG NQFALYMVME
Pigeon               MKPFLVL--AL-C CSFLSSRATP -LPFEFLDCDD PDVFKAVDAA LQEYNGDRASG NQFALYTVVE
W-T Tropicbird       MKLFIVL--AL-C CSFFSSRATP -LPFEFSDCDD PDVFKAVDTA LKKYNGDRGTG NQFALYMVME

Alligator            M---------L-C CLFKSTND                                  DFMLL-----
Green Sea Turtle     MKLSTVL--VL-C CSFFSSRASP -LPTQDADCDD PDVFEAVDIA LRKYNGDKTDG NQFALYMVME
E Brown Snake        MEVFI-LLLLGIGFCQAARDKVDR ---------ND PEVVDAVAGA IAALNEDRSHG NKLALGAILH
Anolis Lizard        MELFILLVLTF-C C----KQAVP -LEGEDADCDS PDVFSAVDLA VKAHNEDQKHG NLFALRVILA

Xenopus leavis       MKHLFSF--IFF- LHLL--RG-S ASQTIEADCND HNIFYAVDEA LRHHNKELIDG NQFVLYRITE
Xenopus tropicalis   MQHLFTF  IVF- LHLL--RG-S ATQAIEADCND HNIFNAVDEA LRHHNRALTDG NKFVLYRITE
Nanorana parkeri     MVV------VVV- VGGDS AAVP V-PDIGVDCDD PNIFKAVDEA LRYYNDAKEDG NQFLLFRV--

Latimeria            MKVLALV--LL-C TKLYVSWAK- -SLLQPADCGD PRVHQAVDAA IKKYNEELKDG HQFALYRITK

Zebrafish            MARDKILTVLA-M LWLYFCGGLA -QTDSSVPCDD RRVEKVVNLT LGTHNKMITEG AQLALYEIL-
Pufferfish           MRSGLGLCVLG-L LCLSSSVR-A -QEPVKVSCDD PSVEKAVSSA VEKFNEKLTTG NKLALFQIQ-
Elephant Shark       MKLFVVLLFSSQLLHSNARSVSDIDSVDPIPIDCDD PELLKAVDFT LRKFNGERRTT HQYALDRVS-


                    44
Human                ATKT-VGSDTF -Y-S-FKY-EIKEG -DCPVQSGKT WQDCEYKDAA KAATGECTAT VGKRSSTKFS
Dog                  GTRT-DDPDTF -Y-S-FKY-QIREG -NCSVQSGKT WQDCDYKEST QAATGECSAT VGKRGKTKFS
Siberian Tiger       VTRT-DDPETF -Y-S-FKY-QIREG -DCSVQSDKT WQDCDYKESA QAATGECSAT VGKRGNTKFS
Grizzly Bear         VVRT-DDPDTF -Y-S-FKY-QIREG -NCSAESGKT WQECDYKDAA QAATGECSAT VGKRGNTKFS
Hippopotamus         VTRM-DNPDTF -Y-S-FKY-QIKEG GDCPVQSNKT WQDCDYKDSA QAATGECTAT VAKRGNMKFS
BN Dolphin           VIRT-DDPDTF -Y-S-FKY-QIKEG -NCPVQSDKT WQDCDYNDSA QAATGECTAT VAKRGNMKFS

Opossum              VSLT-ESSQRT -F-T-VTY-NIQEG -DCHVRMGKN WKECGIKKDL NKERGQCTAI VKSHNENEFT
Koala                ASLT-DSSERT -F-T-ITY-EIRES -NCMIETGKN WKECSYKDSA EWKQGECTAI VKSQNGKEFK
Wombat               TASL-TDSNER -T-FTITYE-IRE- SNCMIETGKN WKECSYKDSA KWEQGECTAI LKSQNGKEFK

Platypus             ASMT-TDSDTF -Y-S-LKYQ-IREG -DCPVQKDKH WQDCDYREAA EAATGECTAT VKTKNKEKFT

Kiwi                 GKKT-AGPDTQ -F-Y-VKYQ-IRET -SCAIEENKH WKDCDYKAPA EAKTGECTAR VHINKAEKTS
Pigeon               AKRT-VGPVTQ -F-H-VKYR-IRET -TCATEENKL WQDCDYKASA EAQTGECIAQ VHLNDAEKTS
W-T Tropicbird       AKKT-ASPDTQ -F-Y-VKYR-IQET -TCAIEENKL WQDCDYKVPS EAKTGECTAR VHMYNTEKTS

Alligator            FFSQ-AGPGAQ -F-F-VKYR-IRES -TCAIGEGKA WQDCDYNAAV EAETGECTAE VYIDKTQKIS
Green Sea Turtle     AKRI-EGSGKQ -F-F-VMYR-IRES -SCAVGGDKL WQDCDYRASA EAESGECTAQ VYVDKTEQIS
E Brown Snake        AYRI-ADPRKK -FLIIYHVR--ETV --CPIAVDKP WQKCELLRTS KAHSGKCTAN IDINESEQFT
Anolis Lizard        ARRT-AGPGKN -F-L-IKYQ-LAET -SCPLKGSVS WQNCEFLPPS EGDSGECTAE IHTDDSQVFS

Xenopus leavis       ARI-KTEN-GG -T-HNFVSYDIREG -SCGVKSGKV WQNCDFKQSD E-KVGKCSAH IVVNKELKTS
Xenopus tropicalis   AKI-KIENDSG -T-HNFVSYDIREG -SCGVKSGKV WQNCDFKQSD E-KVGKCSAH VLVNKELKSS
Nanorana parkeri     TDA-KQRNDEN GQIHYFLDYEIREG -SCTVKSMHS WQDCQFQAHT P-EQGKCSAH LLINTEKKIR

Latimeria            AKTQ-LE-KE- ---THYFVTYEIRE STCSVHDNKI WQECNYVSPI SATTGTCIAE VYIDETVKTS

Zebrafish            ----EATKAQN ESGDVLLVRFSSRE TDCPAGGEKT WHECDYLQQA DKALRICHAK VQFTEAGEEL
Pufferfish           ----SASKTGS GADAVYSLQFTSRR SDCPAGGIKP WTDCDYLPRR -KSPVPCSAI VHVTATEVNT
Elephant Shark       -----FGTVQR KRGSRYFIKFDIQE SNCLVESEKT WTECDHRPPT VANIGHCESS VYIHRAGRIL
```

Supplemental Figure S1. A) Amino acid sequence alignment of vertebrate high molecular weight kininogens (continued).

```
                     103          114
Human                -VATQTCQITP  AEGPVVTAQY  DCLG---CVHPIS TQSPDLEPIL RHGIQYFNNN TQHSSLFMLN
Dog                  -VATQTCQITP  AEGPVVTAQY  DCLG---CVHPIS IASPELEPVL RHAIEHFNNN TDRSHLFALR
Siberian Tiger       -VATQTCQITP  AEGPVVTSQY  DCLG---CVHPIS TASLDLEPVL RHAIQHFNNH TGRSHLFALR
Grizzly Bear         -VATQTCQITP  AEGPVVTAQY  DCLG---CVHPIS TASPDLEPVL RHAIEHFNNN TAHSHLFAVR
Hippopotamus         -VATQTCQITP  AEGPVVTEQY  NCLG---CMHPIS TTHPDLEPVL RHAIQHFNNN TDHSHLFELR
BN Dolphin           -VATQTCQITP  AEGPLVTAQY  DCLG---CLHPIS TESPDLEPVL RHAIQHFNNN TDHSHLFDLK

Opossum              -ITEQHCKIIP  VNDEVIAVNV  PCLG---CYRPIS ANDEDLQAVL NNAVEQFNYQ SQSDHLYTLK
Koala                -VIEQNCHIIP  AHDVVVAVHS  PCLG---CFNPIS TNHSDLEEIL KHGLQSFNEK SKHEYLFALK
Wombat               -VIEQNCHIIP  AHDVVVAVHR  PCLG---CFNLIS TNHSDLEAIL KHALQSFNEK SKHEYLFDLK

Platypus             -VSLQTCQITP  AEGPVITAHY  ECSG---CIHPIS PTSTDLIPIL KHGLQHFNNR TNHPFLFRVN

Kiwi                 -NVSQDCKTVP  ATATIIPAEA  LCLG---CYYPIS SDSLQVSEIL KQAIQKFNRH SDEAALFKLV
Pigeon               -NVSQDCKISP  ATPKVTRTEA  TCLG---CFHPIS SDSSEVSEIL KQAIQKFNRH SAESALFKLV
W-T Tropicbird       -NVSQDCKIFP  AMPKITVTEA  TCLG---CFHPIS SDSSGVSEIL KQAIQKFNRH SAEPALFKLV

Alligator            -NVSQECKIIP  AAGKVTLSQA  PCLG---CYHPIP GNSLDLLPIL RYAIRNFNKE SQKSFLYEVG
Green Sea Turtle     -NVTQECKIIP  VEGKVILSHV  QCLG---CYHPIP GDSLQLLPIL RYAIRIFNNQ SEQSSLFEVG
E Brown Snake        -SISQNCKISQ  GQQNIEQSHV  EFVGLSQCVGCWH RIKTLSPRVL HIVRHTVRQF NNQSQHSSLF
Anolis Lizard        -SVFQTCRITT  APGKVTRSHA  RCLG---CWHTIS SKSEELVPIL RRAIYLFNNE SDQQPLFDAV

Xenopus leavis       EVIIQNCSTFQ V-EPTVSAIKQ  DCLG---CPINLD TRNKDLLPLI KSAIEKMNKL ANYPFYFDLE
Xenopus tropicalis   EVIFQNCSTFK V-EPTVTAVEQ  GCLG---CPIKLD TTNEELLPLI KVAMEKMNKV ANHPFYFNLE
Nanorana parkeri     TVVSQTCSVSK VPLEPYVTAVHH QCLG---CPYPID TNNEEVLRFV HIAIEKMNRQ GSHLYYFDLD

Latimeria            -VVSQKCDLVP -PKDPIVPSVA  QCLG---CPREIP TNSSKVKVVL DAALKKYNKE SNHSFHF---

Zebrafish            --LLHDCLEPA -----IIASVA  PCIVTEECHPLLE KTEVLKCNSS VDVAPWRHEV PEVHVVCEAGVSKTNS
Pufferfish           --RHVECQIDG ----HFTPEKA  PCLG---CEMEID ENSEDLKSPL SVSITKYNSM SNSLHLFTLN
Elephant Shark       EVTMYNCTIIP VNDPWIVPKIA  PCLG---CAISLP HNSSRAKETL DYSIKKFNSD SNYPNIFGSE


                    163
Human                EVKRAQRQVV AGLNFRITYS IVQTNCSKENF LFLTPD-CKSL  -WNGDTGECT DNAYIDIQL
Dog                  EVKKAHRQVV TGWNYEITYS IEQTNCSKENY LFLTPD-CKSL  -LNGDIGECT DHAHMDLQL
Siberian Tiger       EVKRAHRQVV AGWNYEITYS IVQTNCSKEHF LFLTPD-CKSL  -LNGDIGECT DHAHVDLQL
Grizzly Bear         EVKRAHRQVV AGWNYEITYS IAQTNCSKENF LSLTPD-CKSL  -LNGDIGECT DHAHMDLQL
Hippopotamus         XXXXAQRQVV AGWNYEITYS IVQTNCSKENF LFLNPD-CKSL  -PSGDVGECT DKAYVDIQX
BN Dolphin           EVKKAQRQVV AGWNYEITYS IVQTNCSKENF LFLTPD-CKSL  -SNGNIGECT DKAYVDIQL

Opossum              DVLKALRQVV RGWNYDLEFT VVETNCVKSEV KNVTSE-CKPL  -PQGKSMACR ELSHVSPEM
Koala                EVMNASRQVV NGWNYKIQYS IVQTDCSKKED GQLSDK-CKPT  -PQGEISVCS DFTYVDPQM
Wombat               EVMNASRQVV NGWNYKVQYS IMQTDCSKKED GQLSAK-CKPV  -PQGEISVCS DFTHVDPQM

Platypus             EVKKAQRQVV SGWNFDVHYT IIQTNCSKQDF EELLPD-CKPM  -PGGDTGDCS DKAFVDPHM

Kiwi                 EIKEAKRQVV AGWNYIIKYE IKETNCSKDQF QDLSPE-CKTT  -STGRVGNCE AKAYANLND
Pigeon               EIKEATRQVV AGWNYIIKYE VEETNCSKDQF QDLTPE-CKTT  -SRGHIGKCD AKAYVNPQG
W-T Tropicbird       EIKEAKRQVV AGWNYIIKYE IEETNCSKDQF QDLTPE-CKTT  -SRGRVGKCD TKAYENLHA

Alligator            EIIKATRQVV AGWNYAVEYM VKETNCSKKEF QDLSPK-CKPI  -FGGHVGNCV AKAFVDLSN
Green Sea Turtle     EIIKATRQVV AGWNYAVEYE VKETNCTKNNF QDLSPE-CKPI  -VGGHVGRCE AKAYVDLTN
E Brown Snake        GFPVINEAES QVVNGVNYRF KYSINETNCSK KEFLDLSPECR PLSGGLKVFCE AKAYVDNRG
Anolis Lizard        GVVHAARQVV AGWKYKFEYW IQETNCSKADF ADMAPE-CKIL  -PKGHVGSCH VESYVDFRN

Xenopus leavis       NIIEATLQVV AGWNYRLIYT VRQTNCSKSIH SNVPLEECDLD  -ANGQNGTCT TQVFKNTRG
Xenopus tropicalis   TITEATRQVV FGWNYKLFYT IRQTNCSKSIH SNVPLEECNFD  -ANGQNGTCT TQLFINTQG
Nanorana parkeri     QIVNATRQVV GGWDYIINCV VRKTNCSKMDF KTKDSNECKLD  -KEGETGECE LQVSETPDG

Latimeria            EIKRATSQVV AGFKYRVEFR ITETNCSKKDF -EELTEDC-SP  -ISATTGTCI AEVYIDETV

Zebrafish K          RFKRPPGWSP LSKESISPPK HVPLNCPTKPW -KEFKPIIAPP  --NATEPSEP SADTALSDL
Pufferfish           SVGYATRQVV AGFRFKIRFD MKKTTCAKSQH -SDLSDLCVPD  DQNMEFANCN STVDVAPWR
Elephant Shark       VIFKVTSQVV AGYLYTLKFS LRETECTKSSN --DVWQDCILK  PDNATTLYCN STVLFSIRA
```

Supplemental Figure S1. A) Amino acid sequence alignment of vertebrate high molecular weight kininogens (continued).

```
                  222            235
Human             R-IASFSQNCD IYPGKDFVQP PTKICVGCPRD IPTNSPELEE TLTHTITKL NAENNATFYF
Dog               R-IASFSQKCE LFPGEDFVQP PSRICLGCPKK IPVDSPELEV PLTHSIAKL NAENNGTFYF
Siberian Tiger    R-IASLSQKCE LYPGEDFIEP PPSICPGCPKE IPVNSPELEV ALNHSTAKL NAENNGTFYF
Grizzly Bear      R-IASFSQKCD LYPAEDFVQP -SKICSDCPKD IPVNSPELEV ALNHSIAKL NAENNGTFYF
Hippopotamus      R-IASFSQKCN VYPEEDFVAP PTRICAGCPIR IPVDSPELEE PLNHSIAKL NAENNGTFYF
BN Dolphin        R-IASFSQKCD LYPGEDFVQP PTRICAGCPIS IPVNSPELEE PLEHSIAKL NVENNGTFYF

Opossum           K-ISSHLQTCQ --TEADS-QF     SD  MQY ISGYSPELKE SLRAALENF NSENESDFYF
Koala             K-ISIVSQACN --PGSDSASS VT-------QK MSVYSPELKE PLRHSLEKI NSENKNNFYF
Wombat            K-ISIVSQVCN ---------P RSDSGSSVTQK MSIYSPELNE PLRYSLEKI NSESKNNFYF

Platypus          K-ITGFVQNCE LFPGAEWIPP PDLMCAGCPQN LPVDSPELKE PLKHSLDKV NSADNYTFYF

Kiwi              Q-IIDTASQCK FPAEETVDPP -TLICAGCPRP IPKTSPELKE LLEVSMEKY NLETNDDFYY
Pigeon            Q-IVDIASQCK LPVEKTVNPD ---IRTGCTKT IPTDSPELKE LLKLSMEKY NSESNDDHYY
W-T Tropicbird    Q-IVDIASQCK LPVEDTVVPA -TR--TNCPKT IPKDSPELKE LLKVSMEKY NLESDDDFYY

Alligator         T-LVDVTQQCK FPVEETVPPP --QMCPGCPKR IPNDSPELKE VLKASMEKY NSESDDDFYY
Green Sea Turtle  T-IADVAQKCK FPVAETVSPH -ISICAGCPRP IPTNSTELEE PLRATLEKY NAESNDDFYY
E Brown Snake     T-LIHSEVECR PEAEDNMRIL -AQACPGCHSP LAPDSQELKR PLEAVVKLF NIKSSSDFYF
Anolis Lizard     T-IVNVEQKCE LEVDTKKN-- ----CPGCPRT IPSDSPQLKE PLAAIVENY NTKCSNGFLY

Xenopus leavis    E-IIDINLSCF SQ------KG FCLSCPDAVDV DDPELLDLLR QVMDEYNSY NNNTNLYNFV
Xenopus tropicalis E-IKDINLECF SQ------KG FCLSCPDAVDV DDPELLDLLR QVMDEYNSN NNNTNLYNFV
Nanorana parkeri  Q-VNDIILKCT SQAGVC-------LNCPLNVDS DDAELQNLLS QVIDEYNSN INVTNLHKLN

Latimeria         KTSEVVSQKCD LVPPKDPIVP SVAQCLGCPRE IPTNSSKVKV VLDAALKKY NKESNHSFHF

Zebrafish         DLIR
Pufferfish        HELPQVQMECE EGMLIMPLIK RRPPGWTPLRK FEKPGSAAKE ESSEEDTAA AQPSASPVVD
Elephant Shark    NEIDTF-VSCS TDPIGFQMEG FRSQPTETIGI LAQQRQRHRQ HHFYQQQDS HEQHLINKSE
```

```
                  282
Human             KIDNVKKARV QVVAGKKYFI DFVARETTCSK ESNEELTESC ETKKLGQSL DCNAEVYVVP
Dog               KIDSVQSATV QMVAGEKFFI QFVARETMCSK ESNEELAESC QINKYGEQL KCEAEVYVIP
Siberian Tiger    KIDHVKSATV QVVAGKKFSI EFTARETTCSK ESNEELTESC NTNKFGKIL DCKAEVYVIP
Grizzly Bear      KIDSVKSATV QMVAGKKFSI AFIARETTCSK ESNEELTESC QINKYGHTL ECKAEVFVIR
Hippopotamus      KIDMVQKATV QVVAGKKYSI AFTARETTCSK ESNEELTKSC EIHKHGRLL SCNADVYVVP
BN Dolphin        KIDTVEKATV QVVAGKKYSI VFTARETTCSK ESNEELTKSC EINKHSPIL NCKADVYVVP

Opossum           KPSILLKAHL -VEPGEKHSI EIQVQETECSK EKG-QFSEDC EFKTDGRVL QCIVQVPMGQ
Koala             KMETIEKAES PAGPGPKYII EFLIKETECSK EKD-KYSEDC AFKESGDGL KCIANVSVED
Wombat            KMDTIQKAES LVGPEPKYII EFLIKETECSK EKD-KYSEDC TFKELGDGL KCVANIPMED

Platypus          KVETIRKATF QLVAGQKFSI EFLVRQTRCSK EDNEKMPEDC EVDSNGKVL VCNAMVYMVP

Kiwi              KAGDIEKATV QVVAGKSYRI TFTVKKTNCSK KEFEKLNEDC EATPNSVRL KCEAQIYVIP
Pigeon            KSGDIEAAAV QVVSGKIYHL EFAVRKTNCSK KEFEKLHEDC EFTSDSAPL PCEAQIHVIP
W-T Tropicbird    K-GEIEEATV QVVAGQNYHL IFAVRKTNCSK KEFEKLNEDC EATSDSAPL PCEAHVHVIP

Alligator         KVESVFHSTV QVVAGKNYEI EFLIGKTNCSK SEVEKLNEDC KIVIPKISL QCTANIYVVP
Green Sea Turtle  KAEVILYATV QVVAGKNYNI RFKIRKTNCSK TDVKKLNEDC VTTTDSKPL LCTAQVYVIP
E Brown Snake     KIVDITKISG QMLVGHVYRI DFKAQRTNCSK AEVEKPDKNC HAVKGGELM TCHALIYVKP
Anolis Lizard     RITKVTKATV QIVSGIMYRI EFQITETNCSN AEVHELNEDC IAMENSESL QCYGSAWEKP

Xenopus leavis    SVDYASKKGV HEKTYDVTFN ---IKETNCSK SDYAILGEEC QFIETKNAL NCDAKVNVTD
Xenopus tropicalis SVNQASKKGT HEKTYDVKFN ---IQETNCSK SDYSILGEEC EFIETKEAL NCDAKVNITD
Nanorana parkeri  QVIKATKHGF QEQIYEVLFS ---MMPTVCSK PDHTILGDEC NNLENASPL SCDTTIKVTD

Latimeria         GVVEIKRATS QVVAGFKYRV EFRITETNCSK KDFEELTEDC AISEK-NPH NCNSATTVVP

Zebrafish         VVPDDPLHCP SK
Pufferfish        DPLHCPSKVV PD
Elephant Shark    PAVITTSAPL ELPLSAIDQL ADLLGPEPPVN CPGKPWKPL QSN
```

Supplemental Figure S1. Amino acid sequence alignment of vertebrate high molecular weight kininogens (continued).

```
                    342                   358                          384
Human               -WEKKIYPTVN --CQPLGMIS-LM KRPPGFSPFRS SRIGEIKEET TVSPPHTSM --APAQDEERDS
Dog                 -WEKKIYPTVN --CQSLGKVI-LM RRPPGFSPFRS SFMEKTEKGT TVSSPHNSM --VPVQDEEWDS
Siberian Tiger      -WEKKIYPTVH --CQSRGETT-LM KRPPGFSPFRS VQVEKTKEGT TVSPPHTSM --ASVQDEEQDS
Grizzly Bear        -WEEKIYPTVN --CQPLKKII-LM KRPPGFSPFRS VPLEKTEEGT TVSSPHISM --APVPDEERDS
Hippopotamus        -WEEKIYPTVN --CQPLGQTS-LM KRPPGFSPFRS VQVEKTKEGT TVSPPHPSM --APEQDEERDS
BN Dolphin          -WEKKIYPTVN --CQSLGQTT-LM KRPAGFSPFRS VPVEKTKEGT IVSPPHTFL --APVQDEERDS

Opossum             DGEVKPVI--D --CHEPPPELGLM KRPSGFSPFRS AL--RILEEK IIAAREPQN FNTTEQEEEQTP
Koala               --EGTFNPTVR --CEHPTEM--LM KRPPGFSPFRA AAVIPEMEGA EAPSEPQTS -DMTDQEEAQGP
Wombat              --EGTFNPTVH --CEQPTEM--LM KRPPGFSPFRA AALIPETNGA EAPSEPQTS -DTTDQEEAQ-P

Platypus            -WKNEVFPTVT --CQDLEMSSFL- KRPPGFSPFRS VQT-PAKEGS NVSPPQPPK -APDREEE-QA-

Kiwi                -WENKILPQVN --CTEELLPVFLA RRPPGFTPFRT AQYFAQSQPD TTS------ -SNKNETES
Pigeon              -WENKILPQVN --CSIERSAAVLL RRPPGFTPFRS --FVALGQPN ENSCSDQNE KEMQRPGEETG
W-T Tropicbird      -WENKIFPQVN --CSKERSMTVLL RRPPGFTPFRS FAMLSQPS-E IL-CSDKNE EERQTPGKEMR

Alligator           -WKQEIFPQVN --CSEVTPIIQAR RP-PGFTPFRS L-MLHEIYPQ TSPLQTAEE GKDPDKGPRENLGPGLENE
Green Sea Turtle    -WTKTIRPKVS --CAEEENLLMRR PPGFT--PFRS LAVEAKTVQY T-PQIKNEK GPREGQGRGKGSGK
E Brown Snake       -WEPSVVPEVT --CTDDQPFQAHE LEEPNILEDGF NIFHDYEEQK WFLWYLLGRY TLSVILKLF
Anolis Lizard       -WQPKSEVEVT FKCMEKAFNTALL RRPPGFTPFRS AAMATEEN-- TQVCGHRH- GHKNGHNKTKPKSSEDLQE

Xenopus leavis      -TKIIVASSPI --CNARARTMEFL NQFLAKMGDNT IFVVYSAMFT YKGLSPFRG VLPQGSPYRI
Xenopus tropicalis  -TKITVASSPM --CIHRVRST--- -------PFFS ---------- YKGLSPFRM VLPQANPSNK
Nanorana parkeri    -KRINVHSGPV --CVEQQA----- ----------- ------LIMR LSGLSPLRM SKKPDQAETN

Latimeria           -WKNTTTTDVN --CVL---EMALL RLPAGMSPFRV LQATPDSAK- --------- ----------

Zebrafish
Pufferfish
Elephant Shark


                    402
Human               GKEQGHTRRH -DWGHEKQRKH NLGHGHKHE RDQGHGHQRG HGLGHGHEQQ HGLGHGHKFKL
Dog                 GKEQGPTHGH -GRGHEKQIKH GHKYKHDQG YGHNRGHGLG HGHQKQHGLG HGGQRELDFDL
Siberian Tiger      GKEQGPTRGH -GWGHGKQIKH GFGHGHKHE HDQGHRHNRG RGLGHGHQKQ HGFAHGHQKQH GFGHGHQQ
Grizzly Bear        GKEQGPTRGH -GWGHEKQIKH GFGHGHKPE HDQGRGYHGG RGLGHGYQKQ HGLGHGHQREH
Hippopotamus        GKEQGPNRGH -GWGHGNQIKH GLGHGHKXX XXXXXXXXXX XXXXXXXXXX XXXXXXXXXXX
BN Dolphin          GKEQGPTHRH -GWGHGKQIKH GLGHGHKHE HDQGHGHQKG HDLGHGHQRG HGHQGGHGLAH

Opossum             GK-AGYP--H -DHGHGWQRGR HPVHGTKNH PGSGLGHKHG HNHSHGRHRG HDLG-------
Koala               GKEQGLTRSF -GPGH--QKEH NSGHGHK-- ------DHRG LGLGHKHEHA H----------
Wombat              GKEKGLIR-- ----------- SFGHGHRKE HSSGHGHKDH PGLGLGHK---HGH--------

Platypus            GKRRGHPLGH -DSCRGRGPGQ GRGRGCKRA RD-------------------------------

Kiwi                QTPSTETR-- ----------- --------- KDHGHGPEGE GEPGCKHRHK HGCKHGHGFKK
Pigeon              KDGGQEPE-- ----------- ---GEGEPE HKHGHKHEHK HGHKHEHKHE HKHEKDHKPDK
W-T Tropicbird      KDGGQEPEGK GEPEHKHRLMX GRKHRHGYI KDHESDKRHR HEIGCGHRTG YGHGHKKHSKN

Alligator           DGQGDGCDHR VGHGNGHGHGQ GHRFNHKPG HGRGRGRGHD IGRGHKKHQK KDKHKDSK
Green Sea Turtle    DIRHEPA-HK QGHRHDIGHGP EHDHRRGHE DEHGCRHDIG HEPEHEHRRG HGHKDEHRCRH
E Brown Snake       LSFPCSSEAQ RSFSH
Anolis Lizard       DADKHDESLP TASTSNPLKLA MEVFILLVL SVGLCQAGPV QDDVSCDDPE XVFEAVARAIT

Xenopus leavis
Xenopus tropicalis
Nanorana parkeri

Latimeria           -KEEKE---- ---TELRRT- ---------DD KPHGHERGRG RGRGQEHGR GPGHEH----

Zebrafish
Pufferfish
Elephant Shark
```

Supplemental Figure S1. Amino acid sequence alignment of vertebrate high molecular weight kininogens (continued).

```
                  462                                              503
Human                DDDLEHQGGH VLDHGHKHKH GHGHGKHKNKG KK-- NGKHNGWK TEHLASSSE DSTT-PSA---
Dog                  EHQRRHGLGH GHQRGHGLAH GHKYEHGHGHE KY-- KNKRKDNG KNNGRKTEN LAGS-PED---
Siberian Tiger       QHQGRHGLGH GHQRGRGLAH GHTHEHGHGHG KY-- KNKRKDNE KDNGWRIEH LASS-SED---
Grizzly Bear         ---------- ------GLAH GHIREHGQGHG KY-- KNKRKDNG KPNDWKTEH LASS-PEE---
Hippopotamus         XXXXXXXXXX XXXXXXXXXX XXXXXXXXXXX XX---XXXXXXXX XXXXXXXXX XXXXXXXXXXX
BN Dolphin           ---------- --GHKHGHGH GKHKNRGKNNG KH---DGWRTEYL ANFY----E DSTI-SSS---

Opossum              ---------- ---QGNKHKH GQGHWKHEKKP KK---NRK--SWM DEYPYSPTE EN-F-PSS---
Koala                --------SQ GHGHRHKHKH GEGHRKHEKKG KK---SP--GSWT DRYLDSPAK ENS--PS-KT
Wombat               ------AHSQ KHGHRHKPKH GEGHQKHEKKD KK---S--HGSWT NGYLDSPIK ENS--PSSKT

Platypus             ---------- --HERSPECD RGQGKHRNKDR PR---GKPNGGK- -KNCPEDNS NPSR-PEQ

Kiwi                 DHASDKRHRQ G--IDCGHRT GHGCGHQKHSK HGKHKHPNPKSSE ESDERVFNH KETL-PSS
Pigeon               RHRHEIGCGH KTGHKCGHKK HSKNG--KHPN PESSEESDERVFN QDETSPSST DETASPEAAV
W-T Tropicbird       GKRKYPNPKP SDESDERGFN QNKTIPSASAE TDKRHRHEIGCGH RTGYGHGHK KHSKNGKRKYPN

Alligator
Green Sea Turtle     DIGCGSEHE  DKHGRGHGHGK HRIKDKCKHS- TEESSEESHDKVT NQKETLLAV VAEIQSHK
E Brown Snake        AYVDNANTL  VHTQQDCQVQV EEKVGPPVHL  CPGCPVEI
Anolis Lizard

Xenopus leavis       RR-DTERA-- ------KG-- PAHGNKGEQKH VKKHKKEK---KD KRKKKNGHK NEDSSEES
Xenopus tropicalis   NRIDTERA         KRHA HGHGHKEEQKH GRKHKKERKDKKD KKNKKNGHK NGDSSEES
Nanorana parkeri                      LQQH TKGKNKGQHKG RKQEQKGKSDKKR KHKHDDEDE ---SSEEV

Latimeria            ---------- --GHGHKEKK IHGHKQHGDDS SE--EHHSHE ETTVATTIS ESLPDNADKP-

Zebrafish
Pufferfish
Elephant Shark


                            519
Human                ---------- -QTQEKTEGP TPI--P----- --SLAKP-GVTVTFSDFQDSDLI--ATMMPP
Dog                  ----STTSSA -QTQEKTQGP TTL--P----- --SLAQP-GIAVTMPDFQDSDLF--AAVMPN
Siberian Tiger       ----STTSSA -QTQEKTEGP TPL--P----- --PLAQP-GIAVTLPDFQDSDLI--AAVMSN
Grizzly Bear         ----STTSSA -QTQEKTEGP TPL—L------ --SLAQP-GIAVTVPDFQDPDLI--AAVN--
Hippopotamus                                          --SLAQP-GVTVTSPDFQDSDLI--AT
BN Dolphin           ---------- -QVQKKIEGP TPF—-P----- --SLAQP-SVANTFPNLQDSDLV--ATVMPN

Opossum              ---------- -PMQEETQGP PPP--Q----- --SPSQQ-GVDVTPSYFQDFDLLDPNPTNIP
Koala                ---------- ---QEETQGP PPLLSSALQEVT IT-PSDF-QDLDLLDLNPTNPPSEPKTDEKT
Wombat               ---------- ---QEETQGP PPLLSSSLQEVT IT-PSDF-QDLDLLDLNPTSPPSEPTTKEKE

Platypus             ---------- IDPEEPGTPP SILRPTHQPRP EGAVTLSYFRDSDLLSPDTPLAALP------

Kiwi                 ---------- --NAEMVSE- -----LVTPGA VR--------
Pigeon               KKTSSPAECG HKKHSKNGKH PNPESSEESDE RVFNQDETSPSSTDETAS--PEAAV------
W-T Tropicbird       GKRKYPNPKP SDESDERGFN QNKTIPSASAE TASELVNPGVARKKTSTSAEPLILPDISLFNGLPD

Alligator            DKSSEESEEK VLCERESQLP SVDRTSENSQF PTTPSLFQSDALTTPGVTVG
Green Sea Turtle                                                   PDLVELD
E Brown Snake        QQSALYEVRE MKTATRQVVN GWNYNLEYSIK ETNCSKNEFLDLTPECRHLPEGKEGFCTVT
Anolis Lizard

Xenopus leavis       -QEYTILPTV HATQRMQHTT TQTVQLITSAQ KQESLSKTPGEQIS
Xenopus tropicalis   -QEHTILLTQ HTTAQTVELI TST--LTPHSI -----S-TPGGQTP
Nanorana parkeri     -DERG

Latimeria            IPGSGLLPSS VLIKPPSSGP GPVVLPSHPEQ IPAPDKPVTS TVEFPSFPD VALVSASL--

Zebrafish
Pufferfish
Elephant Shark
```

Supplemental Figure S1. Amino acid sequence alignment of vertebrate high molecular weight kininogens (continued).

```
                    558
Human               ISPAPIQ-------SDD DWIPDIQIDP NGLSFNPISDF --PDTTSPKCPG RPWKSVSEI NPTTQMKES--
Dog                 IPPTATE-------SDD DWIPDIQIKP NSLSFNLISDF --PEQTSPKCPG RPWKPVHGM NPTVEVKMN--
Siberian Tiger      TPPTPTE-------IDD DWIPDIQIEK NSLSFNLIPDF --PEKTSPKCPG RPWKPVNGM NPTVEIKEF--
Grizzly Bear        NPSAPKE-------SDD DWIPDIQIEP NSLSFNLIPDF --PEKTSPKCPG RPWKPVNGM NPAVEVKEF--
Hippopotamus        LPPPTE--------SDD DLIPDIQIEP NSLPFELISDF --PETTSPKCPG RPWKPINGV NPTVEMKE---
BN Dolphin          TLPPPTE-------SDD DLIPDIQIEP NSLAFKLIPDF --PETTSPKCPG RPWKPVNGV NPTVEMKEFHD

Opossum             VEPTAEQKTGGEEAEEE VLFPDIPIVP KSPLFTLMPDF PEPEPIVPKCPG SPWQPITVM NPVTEESQNED
Koala               -----KETAGEEEYTDD DWIPDIPIQP KSSLFTLVPDF --PEPPAPKCPG RPWKPIDVM DPVKEESQYMD
Wombat              -----KEKAGEEEDTDD DWIPDIPVQP KSPLFTLVPDF --PEPSAPKCPG RPWKPIDAM GPVKEESPYVD

Platypus            --------------PSD GDLFPEIQSE PKDFSLGLLDF --PEPPPPKCPG RPWKPIQGM DPATEEKQYDD

Kiwi                --------------QET SIPAETVTLP DIFLVNGLPDR --SESPLPRCPG KPWKPIMDL PVPPSFPRELE
Pigeon              --------------KKT SSPAEPLILP DTSFSNGSPDH --PESPLPKCPG KPWKQIMDL PAPDSFPREFT
W-T Tropicbird      ---ASELVNPGVARKKT STSAEPLILP DISLFNGLPDR --PESSLPRCPG KPWKQLMDL PVPPSLPREFK

Alligator           ---PRDDSSTPDIPEEP VSPGTAEIAP DISLFDELPDL --PEPPVSKCPG KPWKSIMQF TNPSENTILFT
Green Sea Turtle    VLPSTLTDGVTEIPDLP AEPDSPGIIP DIPLFGGLPDV --PEPSVPKCPG KPWKPIADL STTTNKPKVLT
E Brown Snake
Anolis Lizard       ---QEDITARSPGEESV GFPSPDSIVP SLSLFERLPDL --PEPPAPKCPG NPWKPIILP PTSLPDPGDFA

Xenopus leavis      KTTEKPTLGLFPHIPSV QEDQDNFFNF HNNAEPDLPGP --DDSNFPKCPG KPWEPVKLP STEPTYDLFDL
Xenopus tropicalis  ETTEEPTLGLFPQIPSV PEDQDNFFNF HDNVEPDLPGP --EDSNLPKCPG NPWEPVKLP ITEPVYNPFDL
Nanorana parkeri    INVNMKEDSHQVLDLPS AQPTVPSKEA VPKDIEEKSNL --ELPYVPKCPG KLWQPRSLT TTVKTFTDDDL

Latimeria           --------------PSF PDVALVSASL PDLHKETFPDL --PEGPEIKCPG QPWKPISPL HGVTSEFSHT

Zebrafish
Pufferfish
Elephant Shark


                                                 617      626
Human                                              YYFDLTDGLS------ ----
Dog                                        PTVEVKEFHDFDLSDALY
Siberian Tiger                                EIKEFHDFDLSDALY
Grizzly Bear                                       PDFDLTDALY
Hippopotamus
BN Dolphin                                           FSLSDALY

Opossum                                              FELSDALSFGKK
Koala                                                FDLSDALEFGKK
Wombat                                               FDLSDALEFGKK

Platypus                                             FDLFDAVR

Kiwi                  DLLPSAVENINPTTENSNPTQNEETSFELSDALQ
Pigeon              DEDLLVFSLKNNDPATESSTSP--QTKDLDLSDALL
W-T Tropicbird      NEDLLTSVKENVNPDTENSTPP--QNKDFDLLDALL

Alligator           NEDLLPNPLENLNPASEKPSPINTDVGDFDLVDALPL
Green Sea Turtle    NEDLLPHLSEDTNPETEKYTLPPQDLDDFNLMDSLL
E Brown Snake       ATDSPELQEPLQNIIESFNANNSGDFHFRIVEIKDATKQV
Anolis Lizard       LEDLLPSEGDVVEPKEISAVAIQPVAADFDLADALY

Xenopus leavis      ASAIGDATPTVAENIENKVPGSTSQG--FNDEDLLLSFI
Xenopus tropicalis  AFAIEDTTTTTTANENIKNKEPGSQGQPFNDEDLLLSLI
Nanorana parkeri    AFAAADFKPLPEKEEEPSKPYTPKQI-PFFDDEDLLL

Latimeria           SSAYEEKSMV GGATDFKDTD LLGF

Zebrafish
Pufferfish          PWKVFNPPSPVAPTDAPNMTADAPVLSDTDLLA
Elephant Shark      RHITPTDPVSETVPPPLNTAEEGEQPKSDGFFFDFDLLAGL
```

B) Cladogram depicting ancestral relationships of extant vertebrates together with LK and HK emergence.

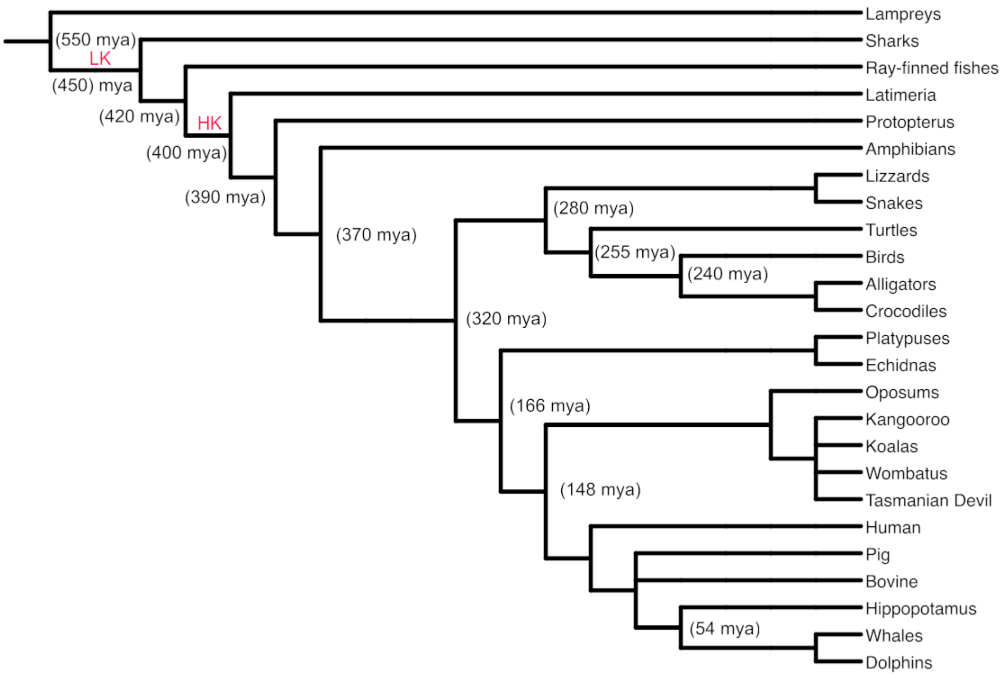

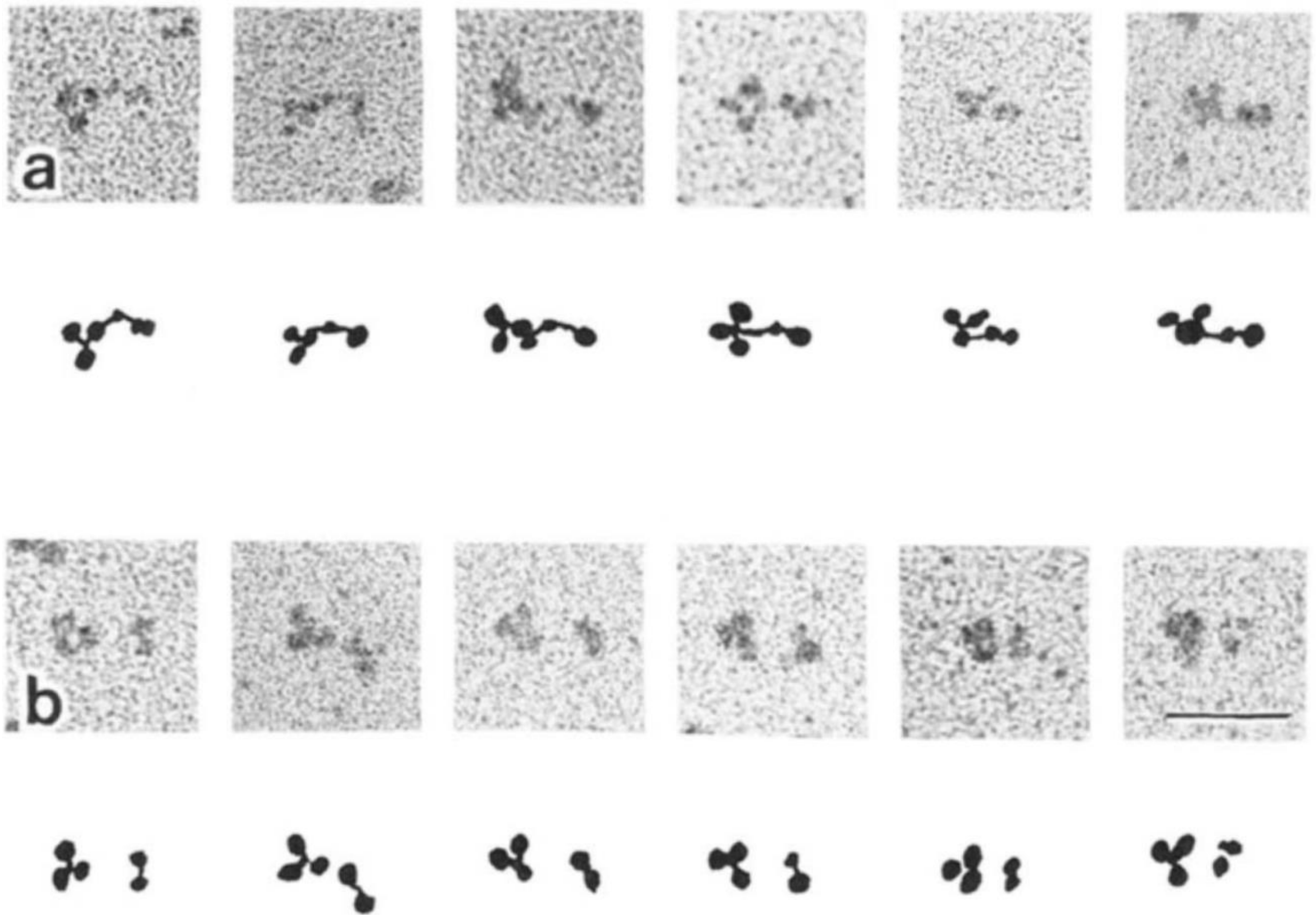

Supplementary Figure S2. Electron microscopy of rotary-shadowed complexes of monoclonal antibody 2B5 with high molecular weight kininogen according to Weisel *et al.* 1994 figure 3 (a) and with cleaved high molecular weight kininogen (b). (Weisel, J.W.; Nagaswami, C.; Woodhead, J.L.; dela Cadena, R.A.; Page, J.D.; Colman, R.W. The Shape of High Molecular Weight Kininogen. Organization into Structural Domains, Changes with Activation, and Interactions with Prekallikrein, as Determined by Electron Microscopy. Journal of Biological Chemistry 1994, 269,
doi:10.1016/s0021-9258(17)36995-8.) The reproduction is presented according to JBC CC-BY license https://www.asbmb.org/journals-news/copyright-and-reproduction.

A

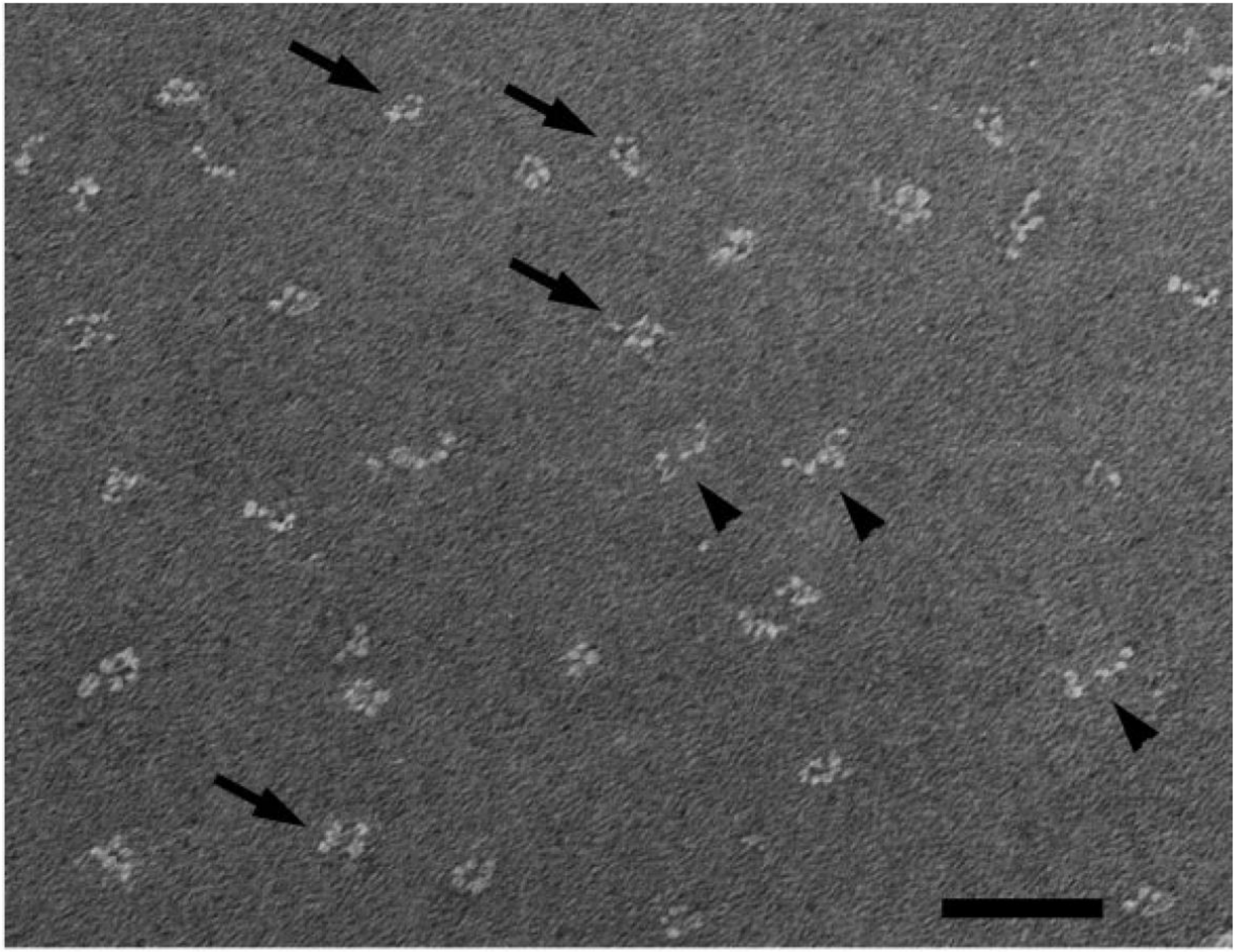

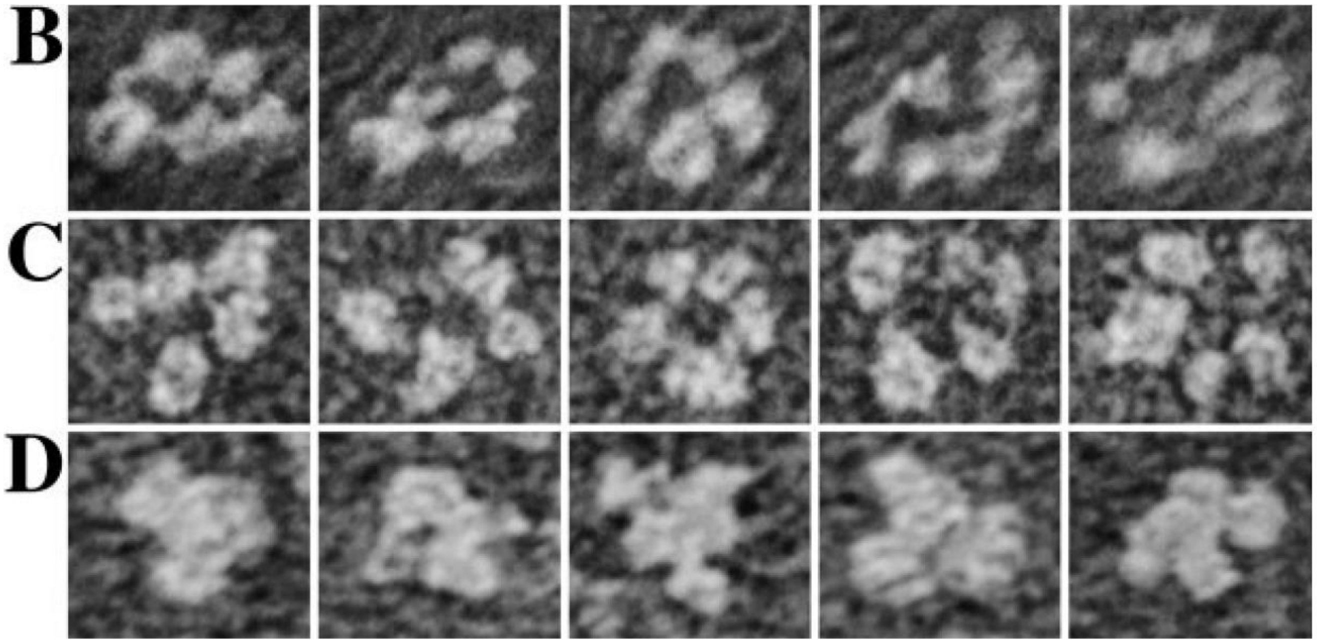

Supplementary Figure S3. Negative staining transmission electron microscopy according to Herwald *et al.* 2001 figure 9 A-D. (Herwald, H.; Mörgelin, M.; Svensson, H.G.; Sjöbring, U. Zinc-Dependent Conformational Changes in Domain D5 of High Molecular Mass Kininogen Modulate Contact Activation. European Journal of Biochemistry 2001, 268, doi:10.1046/j.1432-1033.2001.01888.x.). The reproduction is presented according to FEBS letters Creative Commons License.

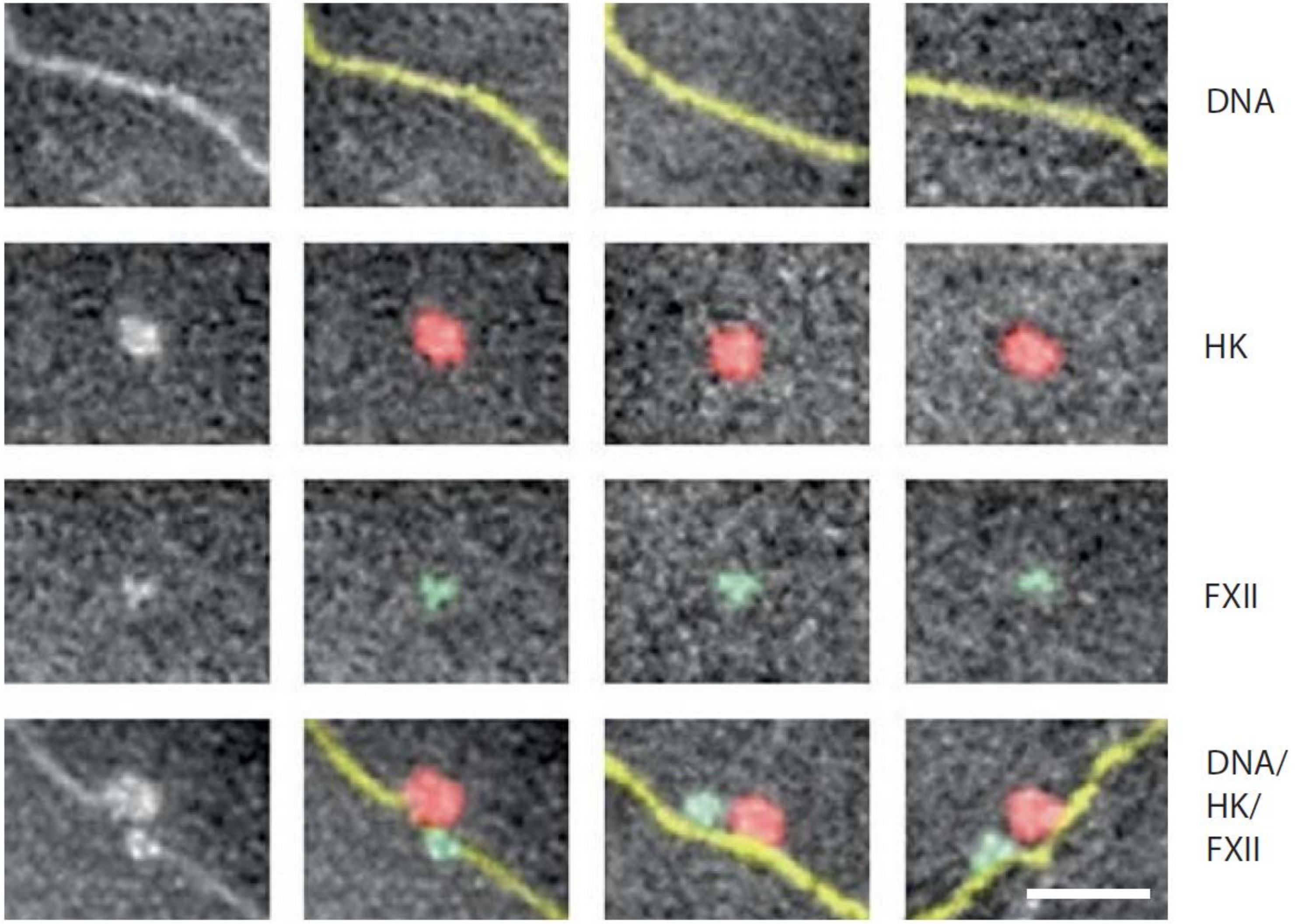

Supplementary Figure S4. Negative staining transmision electron microscopy od DNA and HK figure 1a according to Oehmacke, Mörgelin and Herwald 2009 (Oehmcke, S.; Mörgelin, M.; Herwald, H. Activation of the Human Contact System on Neutrophil Extracellular Traps. Journal of Innate Immunity 2009, 1, doi:10.1159/000203700.). The reproduction is presented according to reuse permission of the figure as MDPI an Karger, are signatories of the STM permission guidelines.
